# Case Report: Nirogacestat therapy induces rapid response in a patient with refractory, life-threatening desmoid tumor

**DOI:** 10.3389/fonc.2025.1714475

**Published:** 2025-11-26

**Authors:** Dennis Christoph Harrer, Markus Herrmann, Martin Vogelhuber, Sebastian Geis, Daniel Mahr, Maya Niethard, Felix Keil, Gerardo Napodano, Wolfgang Herr, Matthias Grube

**Affiliations:** 1 Department of Internal Medicine III, Hematology and Oncology, University Hospital Regensburg, Regensburg, Germany; 2 Center of Plastic, Hand and Reconstructive Surgery, University Hospital of Regensburg, Regensburg, Germany; 3 Dept. of Trauma Surgery, University Hospital Regensburg, Regensburg, Germany; 4 Institute of Pathology, University Regensburg, Regensburg, Germany; 5 Department of Radiology, University Hospital Regensburg, Regensburg, Germany

**Keywords:** desmoid, tumor, nirogacestat, therapy, sarcoma

## Abstract

**Background:**

Desmoid tumor (DT) is a rare soft tissue neoplasm characterized by locally aggressive infiltration into adjacent tissues with few established treatment regimens. So far, the evidence-based therapeutic arsenal for DT has comprised surgery, locoregional therapy, tyrosine kinase inhibitor treatment, and chemotherapy. The γ-secretase inhibitor nirogacestat was approved by the FDA in 2023 and, in 2025, received European authorization as the first and only agent for treatment of progressive DT requiring systemic therapy predicated on positive results from an international phase 3 clinical trial.

**Methods:**

Single case study and review of the literature.

**Case presentation:**

We report on a 29-year-old patient with a recurrent DT located below the right mastoid process with extensive infiltration into cervical tissue and compression of the right internal jugular vein. Following early relapse to surgery, the patient sequentially failed treatment with pegylated liposomal doxorubicin (PLD), sorafenib, and a combination of doxorubicin and dacarbazine. Against the backdrop of life-threatening circumstances due to pending infiltration of the right carotid artery, the patient was subjected to nirogacestat treatment as part of a compassionate use program. Upon four months of nirogacestat treatment, MRI-imaging revealed a sizable regression of the DT, substantially decreasing the danger of carotid artery infiltration. Currently, the patient continues nirogacestat treatment, and no severe side effects were observed.

**Conclusion:**

Albeit rarely lethal in general, DT can exert life-threatening danger by local infiltration into vital tissue, such as blood vessels. The presented case highlights the novel γ-secretase inhibitor nirogacestat as a highly effective therapy preventing infiltration of the right carotid artery by a remarkably refractory DT.

## 1 Introduction

Desmoid tumor (DT), also known as aggressive fibromatosis or desmoid-type fibromatosis, is a slow-growing soft tissue neoplasm resulting from monoclonal fibroblast proliferation within the connective tissue (1). With an annual incidence of approximately 3 to 5 subjects per million, desmoid tumors constitute a rare tumor entity with a preponderance of mid-age patients (median age 30 to 40 years) and a female predominance (2). While the majority of cases occur sporadically, in patients with familial adenomatous polyposis (FAP) the disease occurs in 10–20% of cases (3). DT can stir significant morbidity by locally aggressive growth with infiltration into surrounding tissue and compression of adjacent structures (2). However, owing to absence of metastatic spread, the overall mortality reported for patients with DT usually remains low (2).

To date, few consensus management guidelines have been established for the treatment of DT (4). Historically, complete surgical resection was regarded as the primary treatment option for patients with DT. However, recent prospective studies have demonstrated that active surveillance of newly diagnosed DT is an effective strategy for selected patients with resectable tumors (e.g., small size, asymptomatic, and tumors located in areas where growth does not affect surgical outcomes or cause functional impairment), thus sparing patients from unnecessary aggressive treatments (5–7). Though, in the presence of persistent tumor progression or escalating symptoms, initiation of active treatment is warranted. Standard therapeutic approaches encompass surgery, tyrosine kinase inhibitors (TKI, e.g., sorafenib and pazopanib), low-dose or conventional chemotherapy (e.g., methotrexate and/or vinorelbine, pegylated liposomal doxorubicin (PLD), or doxorubicin combined with dacarbazine), and radiotherapy, with treatment selection largely guided by tumor location (4, 8). In selected cases, cryoablation may also be considered for patients with smaller DTs. However, this technique is not yet widely used (4). In addition, isolated limb perfusion (ILP) has been recently reported as a potential treatment option for DT of the extremities (9).

Surgery for DT is mainly reserved for cases with significant, documented progression that threaten organ function or life, especially in the abdominal wall or parietal locations after failure of active surveillance. However, resection can be challenging in the vicinity of vital structures, such as blood vessels, or in the case of extensive infiltration into surrounding tissue (4). Moreover, recurrence rates of DT following surgery exceed 50% (4).

A hallmark of DT encompasses alterations in the Wnt/β-catenin pathway, with the more frequent sporadic-type DT harboring CTNNB1 mutations and the less frequent DT being associated with FAP characterized by mutations in the APC gene (10). Apart from augmented expression of β-catenin resulting from aberrant Wnt/β-catenin pathway signaling, the growth of DT is propelled by the amplified expression of the oncogene Notch 1. Upon ligand binding, Notch receptors are cleaved by membrane-bound γ-secretases, resulting in the initiation of transcriptional activity by the released intracellular Notch 1 domain, which in turn drives proliferation of DT cells (11).

Recently, a phase 3, international, double-blind, randomized, placebo-controlled trial (DeFi) evaluated the selective γ-secretase inhibitor nirogacestat in adults with refractory DT. It has been demonstrated that treatment with nirogacestat significantly improved progression-free survival, objective response, and quality of life outcomes (12, 13). The results of this landmark clinical trial corroborated data obtained from smaller phase 1 and 2 trials, resulting in FDA approval for nirogacestat as treatment for patients with progressing DT who require systemic treatment in 2023 (14, 15). Just recently, in August 2025, nirogacestat was granted European approval as the first and only agent for treatment of progressive desmoid tumors requiring systemic therapy. In order to treat patients effectively, it is beneficial to share clinical experience in the use of nirogacestat for this rare disease.

## 2 Case presentation

**Figure 1:**
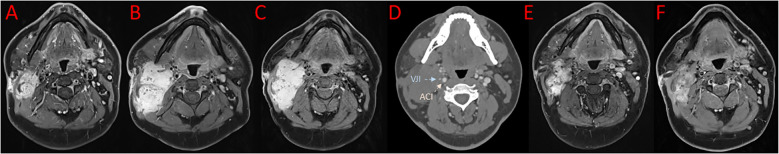
Images in axial reconstruction at different timepoints. **(A)** MRI imaging showing early recurrence of desmoid tumor after surgery. **(B)** MRI imaging two months into sorafenib treatment. **(C)** MRI imaging after three cycles of doxorubicin in combination with dacarbazine. **(D)** Corresponding CT-scan revealing full compression of the right internal jugular vein (VJI, blue arrow) and imminent contact to the right carotid artery (ACI red arrow). MRI imaging after four **(E)** and seven **(F)** months of nirogacestat treatment.

**Figure 2:**
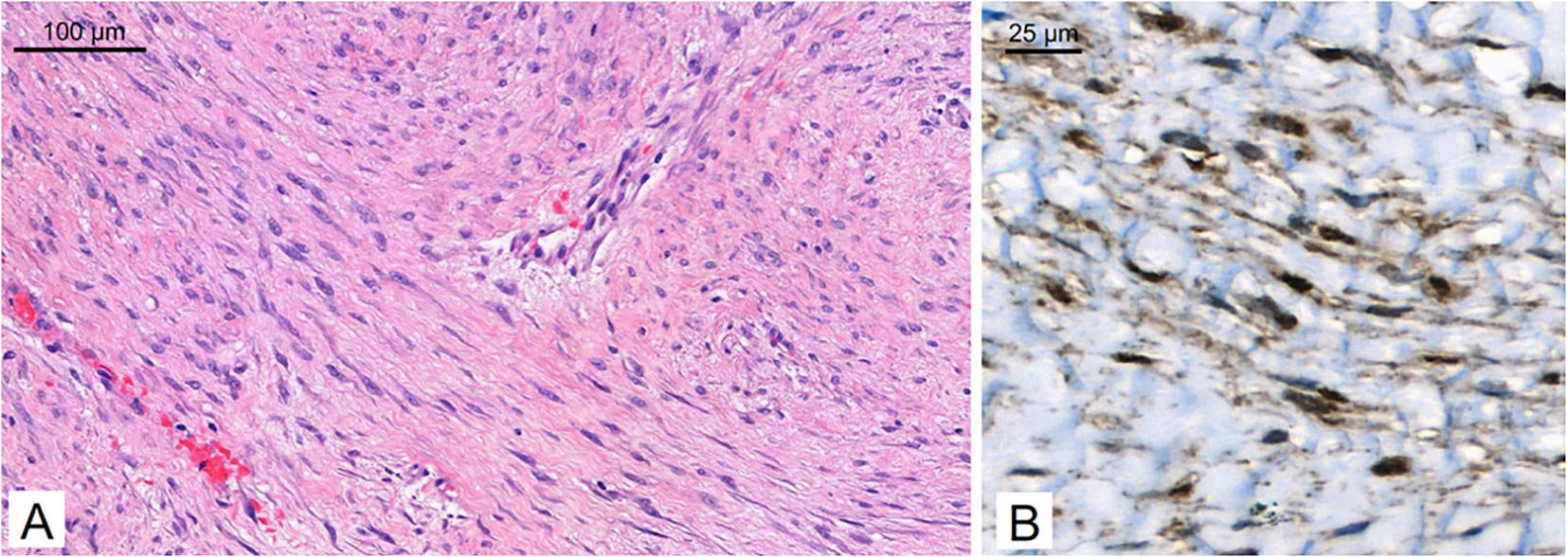
Histopathology. The tumor consists of long, sweeping fascicles of bland spindle cells [**(A)** hematoxylin & eosin] and demonstrates aberrant nuclear expression of ß-catenin on immunohistochemistry **(B)**.

**Figure 3:**
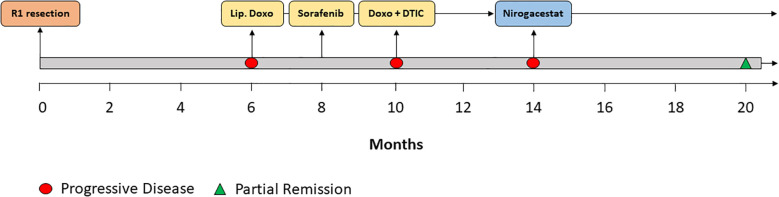
Therapy timeline. Schematic depiction of the different treatments in chronological order. Lip. Doxo = liposomal pegylated doxorubicin. Doxo + DTIC = doxorubicin combined with dacarbazine.

A 29-year-old patient reported a progressively growing indolent tumor below the right mastoid. Ultrasound and MRI-imaging revealed a 26 mm x 35 mm x 40 mm mass adjacent to the right parotid gland with dorsal sound extinction and enlarged cervical lymph nodes. On the suspicion of neoplasia, the tumor was surgically excised at a community hospital. Histopathological examination revealed a DT with a microscopically positive resection margin (R1 resection). MRI-imaging conduced six months later as part of the regular follow-up showed an extensive mass interposed between the right sternocleidomastoid muscle and the carotid sheath, suggesting early recurrence of the DT (**Figure 1A****),** and the patient was referred to our university sarcoma centre. The patient was in good general condition, reporting only a localized sensation of tension in the right cervical region. Owing to size and infiltration into the carotid sheath, the recurrent DT was deemed unresectable, and preparations for systemic treatment were initiated. Against the backdrop of a previous history of hereditary severe precapillary pulmonary hypertension (BMPR-2 mutation), first-line treatment with PLD (40mg/m2 intravenously every 4 weeks) was initiated due to the improved toxicity profile. However, on account of repeated severe allergic infusion reactions despite prophylactic treatment with high-dose dexamethasone and histamine receptor antagonists, treatment with PLD had to be aborted prematurely after two cycles. Next, oral treatment with the TKI sorafenib (400mg daily orally) was started. Nevertheless, MRI-imaging performed two months into sorafenib treatment demonstrated progression of the DT with compression of the right internal jugular vein, and imminent infiltration of the right carotid artery (**Figure 1B**). At this point, the patient remained in good general condition; however, he reported progressive swelling in the right facial region. Given the proximity to the carotid artery and corresponding life-threatening implications, treatment was switched to doxorubicin in combination with dacarbazine, irrespective of pulmonary hypertension. After three cycles of chemotherapy, which were well tolerated without impairment of ventricular function, MRI-imaging once again indicated progression of the DT with now full compression of the right internal jugular vein and imminent contact to the right carotid artery (**Figure 1C**). Clinically, the patient reported a progressive sensation of tension on the right side of his neck, accompanied by increasing swelling in the right facial region. Corroboration for the risk of a pending life-threatening arrosion of the right carotid artery was provided by Doppler ultrasound and CT-angiography (**Figure 1D**). Due to the highly refractory growth pattern, usually uncommon for DT, another biopsy was taken and subsequent histopathological examination reconfirmed the diagnosis of a DT with characteristic aberrant nuclear expression of ß-catenin (**Figure 2**). To explore potential therapeutic targets, an additional biopsy was subjected to whole-genome sequencing. This analysis revealed a CTNNB1 mutation characteristic of desmoid-type fibromatosis (c.134C>T, p.S45F), which is recognized as an independent adverse prognostic factor. No actionable therapeutic targets were identified. On the cusp of an imminent life-threatening arrosion of the right carotid artery, therapy was started with the oral FDA-approved selective γ-secretase inhibitor nirogacestat (150 mg twice daily). With EMA approval still pending at that time, nirogacestat was administered within a compassionate use program (CUP). MRI-imaging conducted at four and seven months after initiating nirogacestat therapy revealed a sizable regression of the DT without any further direct contact to the carotid sheath (**Figures 1E, F**). All previously reported symptoms had fully resolved. No significant side effects were observed by treatment with nirogacestat. As of now, treatment with nirogacestat is intended until disease progression. A schematic portrayal summarizing the different treatments in chronological order is shown in **Figure 3**.

## 3 Discussion

For patients with DT therapy standards are still emerging, and treatment management varies among clinicians (16). Due to the location in the head/neck region and the infiltration of the right carotid sheath, tumor resection or other local ablative therapy was not appropriate for our patient. Therefore, systemic therapy was indicated. Potential options for systemic therapy include low-dose and conventional chemotherapy, kinase inhibitor treatment, and since recently γ-secretase inhibition via nirogacestat (17). Notch signalling is proposed to act synergistically with the Wnt/β-catenin pathway to sustain proliferative and survival signalling in DT through cooperative transcriptional mechanisms, reciprocal regulation of downstream target genes, and direct molecular crosstalk between the two pathways. Pharmacological inhibition of γ-secretase blocks Notch activation and, owing to the interconnectedness of these signalling cascades, also modulates Wnt/β-catenin pathway activity (12, 18–20).

Given that nirogacestat was not yet approved in Europe when treatment was indicated and was only available through a CUP after at least one prior systemic therapy, chemotherapy was chosen as the therapeutic approach. In cases in which swift tumor reduction is required, chemotherapy constitutes an attractive choice. Chemotherapeutic strategies encompass e.g. regimens combining methotrexate with vinblastine or vinorelbine, as well as conventional-dose protocols based on anthracyclines, such as doxorubicin/dacarbazine and PLD (4, 10). Given the presence of severe pulmonary hypertension, first-line therapy was initiated with PLD, owing to its more favorable toxicity profile and reduced cardiotoxicity compared with conventional formulations (21). Multiple retrospective studies and case series demonstrate that PLD offers good tumor control and symptom relief, with objective response rates (ORR) between 30% and 50% across cohorts (22–25). In a large institutional review of 61 patients (26 treated with PLD), 21 of 23 evaluable patients (91%) showed clinical benefit, defined as tumor shrinkage, symptom improvement, or durable stable disease (26). Recently, response rates of approximately 35% were demonstrated in two uncontrolled case series of patients who received PLD (22, 23). However, due to allergic side effects, treatment with PLD had to be discontinued, and treatment with the TKI sorafenib was initiated. TKIs can be used effectively to treat progressive or symptomatic desmoid tumors. Kinase inhibitor treatment is predicated on blockade of signaling cascade triggered by platelet-derived growth factor (PDGF) and vascular endothelial growth factor (VEGF). Sorafenib and pazopanib are widely used for the treatment of progressive or symptomatic DT. However, sorafenib is more extensively studied with a larger patient base, while pazopanib shows similar efficacy in smaller cohorts. Both drugs have manageable toxicity profiles, with some patients requiring dose modifications. Sorafenib mediated a 2-year PFS of 81% and an objective response rate of 33% in the phase III randomized, placebo-controlled ALLIANCE trial (27). While the kinase inhibitor imatinib yielded lower response rates relative to sorafenib, the randomized phase II trial DESMOPAZ evaluating the therapeutic activity of pazopanib showed similar clinical benefit in patients with DT (28). Nevertheless, reported objective responses mediated by chemotherapy have been higher as compared to kinase inhibitors, rationalizing the use of sorafenib as second-line treatment in the present case. Unfortunately, after two months of sorafenib therapy, follow-up MRI revealed disease progression characterized by tumor enlargement with compression and impending invasion of the cervical vascular structures. Furthermore, the patient presented with clinical symptoms resulting from cervical blood vessel compression. Given the critical situation and the necessity for prompt tumor reduction, another course of chemotherapy was initiated using a combination of conventional doxorubicin and dacarbazine. Although associated with increased toxicity, doxorubicin-containing therapies demonstrate higher response rates in DT compared with non-anthracycline protocols. Various studies have demonstrated the effectiveness of doxorubicin/dacarbazine combination therapy in DT, with the largest published series showing an objective response rate of 66% (two out of nine patients in complete remission, four in partial remission) (29–31). Despite combination chemotherapy, the disease demonstrated ongoing progression with concomitant deterioration of clinical symptoms.

DTs refractory to chemotherapy and kinase inhibitor treatment are very rare and pose a jeopardy in case of infiltration into vital structures, such as blood vessels. Given the unusual lack of response to previous treatment, the diagnosis of desmoid tumor was re-confirmed via biopsy and histological examination. Given the tumor dimensions and its adjacency to the right carotid artery, local interventions such as resection, radiotherapy, or cryoablation remained unfeasible. Data on immunotherapy in patients with desmoid tumors are scant, and overall response rates are low (32).

Based on recent results from the phase 3, international, double-blind, randomized, placebo-controlled DeFi trial evaluating nirogacestat in patients with progressing DT, we enrolled our patient in a CUP to enable treatment with nirogacestat. In aggregate, nirogacestat was reported to achieve objective responses in 41% of patients, with 7% being complete responders, and an overall reduction of disease progression or death by 71% (12, 13). Importantly, the study population in this landmark phase 3 clinical trial on nirogacestat was similar to our patient, with a median age of 34.0 years, and the majority of patients (74%) enrolled following an average of two lines of pretreatment, including chemotherapy and sorafenib (12, 13). After four months of nirogacestat therapy, the patient exhibited a marked reduction in tumor size accompanied by complete resolution of clinical symptoms.

## 4 Conclusion

The presented case highlights the potential of the γ-secretase inhibitor nirogacestat as a highly effective therapy preventing life-threatening infiltration of the right carotid artery by a remarkably refractory desmoid tumor.

## Data Availability

The original contributions presented in the study are included in the article/supplementary material. Further inquiries can be directed to the corresponding author.
